# Assessing resolvability, parsability, and consistency of RDF resources: a use case in rare diseases

**DOI:** 10.1186/s13326-023-00299-3

**Published:** 2023-12-05

**Authors:** Shuxin Zhang, Nirupama Benis, Ronald Cornet

**Affiliations:** 1 Department of Medical Informatics, Amsterdam UMC location University of Amsterdam, Meibergdreef 9, Amsterdam, The Netherlands; 2 Amsterdam Public Health, Methodology & Digital Health, Amsterdam, The Netherlands

**Keywords:** Rare disease, Quality assessment, Linked data, RDF

## Abstract

**Introduction:**

Healthcare data and the knowledge gleaned from it play a key role in improving the health of current and future patients. These knowledge sources are regularly represented as ‘linked’ resources based on the Resource Description Framework (RDF). Making resources ‘linkable’ to facilitate their interoperability is especially important in the rare-disease domain, where health resources are scattered and scarce. However, to benefit from using RDF, resources need to be of good quality. Based on existing metrics, we aim to assess the quality of RDF resources related to rare diseases and provide recommendations for their improvement.

**Methods:**

Sixteen resources of relevance for the rare-disease domain were selected: two schemas, three metadatasets, and eleven ontologies. These resources were tested on six objective metrics regarding resolvability, parsability, and consistency. Any URI that failed the test based on any of the six metrics was recorded as an error. The error count and percentage of each tested resource were recorded. The assessment results were represented in RDF, using the Data Quality Vocabulary schema.

**Results:**

For three out of the six metrics, the assessment revealed quality issues. Eleven resources have non-resolvable URIs with proportion to all URIs ranging from 0.1% (6/6,712) in the Anatomical Therapeutic Chemical Classification to 13.7% (17/124) in the WikiPathways Ontology; seven resources have undefined URIs; and two resources have incorrectly used properties of the ‘owl:ObjectProperty’ type. Individual errors were examined to generate suggestions for the development of high-quality RDF resources, including the tested resources.

**Conclusion:**

We assessed the resolvability, parsability, and consistency of RDF resources in the rare-disease domain, and determined the extent of these types of errors that potentially affect interoperability. The qualitative investigation on these errors reveals how they can be avoided. All findings serve as valuable input for the development of a guideline for creating high-quality RDF resources, thereby enhancing the interoperability of biomedical resources.

**Supplementary Information:**

The online version contains supplementary material available at 10.1186/s13326-023-00299-3.

## Introduction

**Fig. 1:**
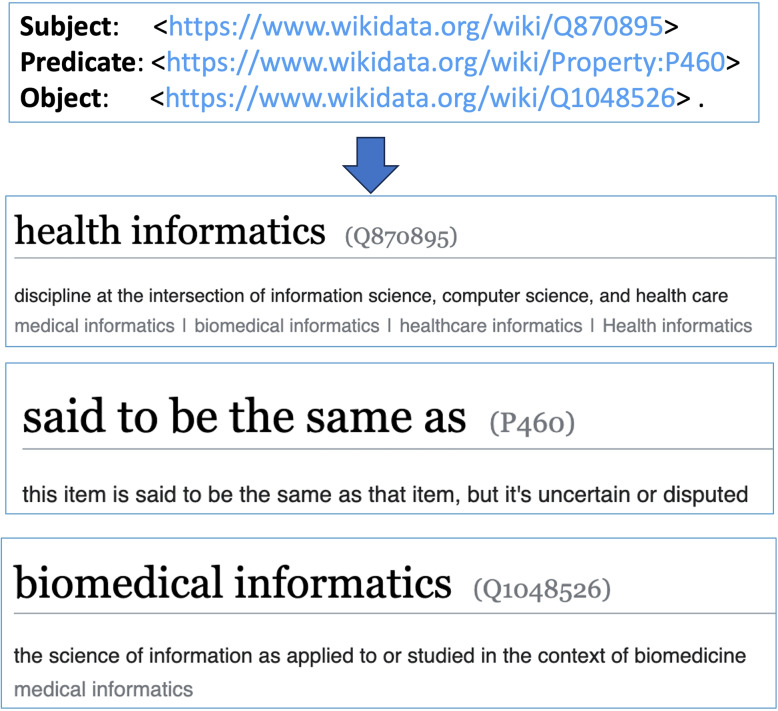
An example RDF triple from Wikidata

The acquisition and comprehension of health data and knowledge are crucial for improving the quality of care for patients. Health data enables healthcare providers to obtain a complete picture of a patient’s health status. It is considered to be useful to represent data and knowledge on the web in Resource Description Framework (RDF) to make them Findable, Accessible, Interoperable, and Reusable (FAIR) [1–5], so that each resource can be identified by a Unique Resource Identifier (URI) and can have qualified references to other resources. Data and knowledge in RDF exist in the form of triples: subject, predicate, and object [6]. Figure 1 shows an example of an RDF triple from Wikidata [7]: the subject ‘health informatics’, the predicate ‘said to be the same as’, and the object ‘biomedical informatics’. Each component is identified by a unique URI with a definition.

This feature enables resource integration in a more meaningful and seamless manner. However, quality issues in the RDF representation of resources can hamper this advantage of RDF data. For example, the URI <http://www.ebi.ac.uk/efo/definition> is used as a predicate of a triple in the Orphanet Rare Disease Ontology (ORDO) resource to provide definitions of its subjects; however, this URI is not resolvable (i.e., it returns HTTP status code 404), making it impossible to retrieve the information (e.g., descriptions) of the resource that the URI points to in a machine-readable manner. Without this definition, it is also impossible to automatically determine whether it is a property or a class. It cannot be semantically distinguished from the property ‘iao:definition’ (http://purl.obolibrary.org/obo/IAO_0000115), another property that is also used to provide definitions as described in the Information Artifact Ontology (IAO). As this example demonstrates, the use of non-resolvable URIs can hamper the ability to provide additional semantics, reduce the quality of the resource, and hence should be avoided.

A set of six foundational metrics [8] was proposed to determine whether an RDF resource possesses the necessary characteristics to maximize the benefits of using RDF. These metrics were selected from a pool of existing measures specific to linked data quality assessment, and were regarded as the minimal quality requirement for an RDF resource to meet. The six metrics, which are represented in RDF at <http://purl.org/fqm#>, reflect three dimensions: resolvability, parsability, and consistency.

In the domain of rare diseases, the added value of making health resources linkable and semantically interoperable is more important, compared to the domain of common diseases, where sufficient data is often available for analysis. In Europe, a disease is considered ‘rare’ when its prevalence is less than 5 per 10,000 people [9]. Such rarity makes it hard to collect, store, and analyze sufficient data for the research and development of treatment. The use of linked resources and RDF can improve the collection and storage of rare-disease data through standardization and integration, which has already been advocated and facilitated by the European Joint Programme on Rare Diseases (EJP RD) [10], an international initiatives in the rare-disease domain. In practice, there are numerous RDF resources related to rare diseases, but it is unknown whether their ‘linked data’ benefits have been fully exploited.

Therefore, we aim to assess the quality of existing RDF resources relevant in the domain of rare diseases, according to the six foundational metrics, and to provide recommendations for the creation of high-quality RDF resources, specifically in the domain of rare diseases.

## Methods

In this section, we describe the process of selecting RDF resources for rare diseases, introduce the metrics used, and describe the workflow for quality assessment and the quality model for structuring the assessment report.

### Materials

We searched for existing rare-disease resources through:

- An EJP RD resource map. It exhibits the various resources that make valuable contributions to the rare-disease domain and collaborate with the EJP RD: https://resourcemap.ejprarediseases.org/.
- A list of FAIR implementations. Data stewards in the EJP RD [11, 12] use this document to record the implementation status of the various resources related to rare diseases.

From these resources, we selected those for which an RDF representation exists.

All the experiments mentioned in this paper were conducted on a MacBook Pro with a 2.3 GHz 8-Core Intel Core i9 processor and 16 GB 2400 MHz DDR4 memory.

### Quality metrics

We used the six metrics to assess the quality of RDF resources on rare diseases, see their definition in Table 1. These metrics are objective, automatable, and foundational [8].

**Table 1:** Metrics as minimal requirements for quality assessment on RDF resources. Modified from [8]

Metric	Definition
Non-resolvable URIs	Measure the proportion of unique non-resolvable URIs to all unique URIs in an RDF resource. A URI is non-resolvable if it returns an error code (e.g., http 404).
Non-parsable URIs	Measure the proportion of unique non-parsable URIs to all unique URIs in an RDF resource. A URIs is non-parsable if its media type is indicated as RDF content-type, but its content cannot be parsed as RDF triples.
Undefined URIs	Measure the proportion of unique, undefined URIs to all unique URIs in an RDF resource. A URI is considered as undefined if it does not exist within the parsed RDF triples resulting from resolving the URI.
Misplaced classes or properties	1) Measure the proportion of classes which are incorrectly used as a predicate to all unique classes; or 2) measure the proportion of properties which are incorrectly used as a class to all unique properties.
Misuse of owl:DatatypeProperty or owl:ObjectProperty	Measure the proportion of misused ‘owl:DatatypeProperty’ (or ‘owl:ObjectProperty’ ) properties to all properties.
Use of deprecated classes or properties	Measure the proportion of deprecated classes or properties to all unique classes or properties.

### Workflow for quality assessment

**Fig. 2:**
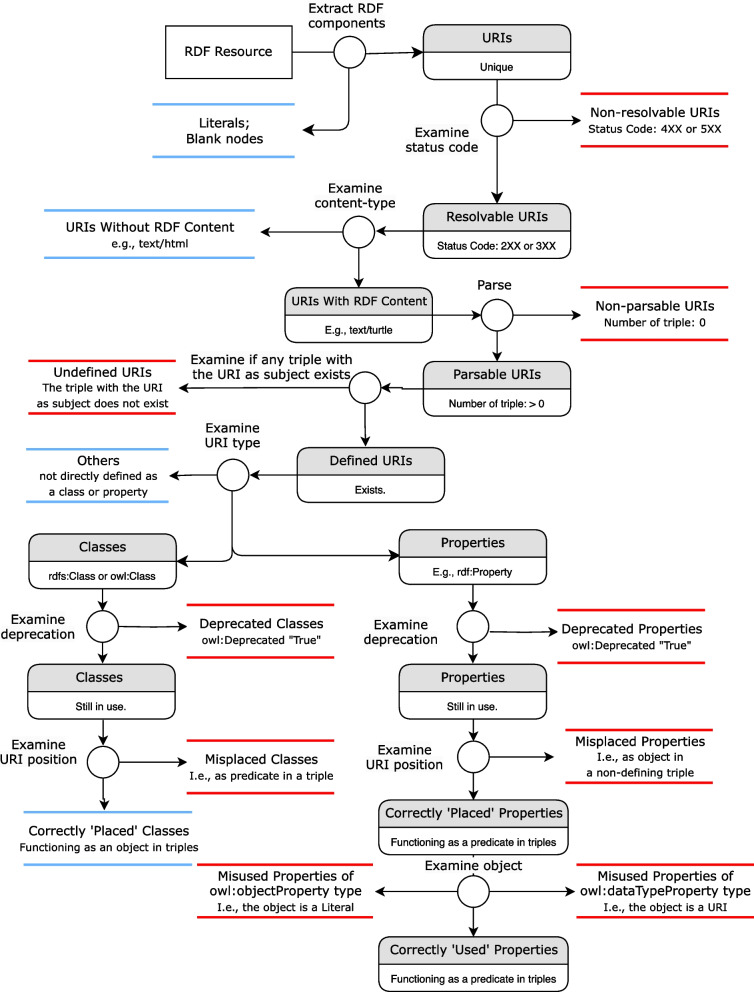
The workflow for quality assessment of RDF resources. The components outlined in red are the errors that fail the quality metrics. The components outlined in blue are those that pass the test

The workflow of quality assessment consists of the following steps aligned with [8], comprise the quality assessment procedure (see Fig. 2):

1. The components of RDF resources, namely URIs, literals, and Blank nodes [6], are extracted. The set of unique URIs is analyzed.
2. The HTTP status codes for URIs are retrieved, and URIs with the status code as ‘4xx client error’ or ‘5xx server error’ are classified as non-resolvable (the ‘non-resolvable URIs’ metric).
3. The content-types of resolvable URIs are retrieved and used to categorize them as URIs that have or do not have content-type RDF. RDF content-type is the Media Type [13] that corresponds to any RDF serialization format, see Table 2. For example, the media type ‘text/turtle’ corresponds to the ‘Turtle’ serialization format of RDF. A URI that does not have content-type RDF is not further analyzed while the content of a URI with RDF content-type is parsed and examined. If the content does not contain at least one RDF graph (i.e., any RDF triple), this URI is classified non-parsable (the ‘non-parsable URIs’ metric). During content negotiation, the higher factor weighting is assigned to the Media Type in Table 2 indicating various RDF serialization formats; the lowest factor weighting is assigned to ‘*/*’, indicating that any other Media Type is enabled if all RDF-related Media Types are unavailable.
4. For every parsable URI its specification is extracted from the parsed graph. If no such specification exists, the URI is classified as an undefined URI.
5. The types of all defined URIs are extracted to identify ‘classes’ (i.e., those of type ‘owl:Class’ or ‘rdfs:Class’) and ‘properties’ (those of ‘rdf:Property’ or any OWL property).
6. The deprecation of each class and property is examined. A class C is deprecated if one of these triples exists:
  $$\begin{aligned} \begin{array}{c} \text {C owl:deprecated "true"}{\wedge \wedge }\text {xsd:boolean .}\\ \text {C rdf:type owl:DeprecatedClass .} \end{array} \end{aligned}$$
  A property P is deprecated if one of these triples exists:
  $$\begin{aligned} \begin{array}{c} \text {P owl:deprecated "true"}{\wedge \wedge }\text {xsd:boolean .}\\ \text {P rdf:type owl:DeprecatedProperty .} \end{array} \end{aligned}$$
7. The role of classes in the triples is assessed. A class is misplaced, if it is used as the predicate of a subject-predicate-object triple. (the ‘misplaced classes or properties’ metric).
8. The role of properties in the triples is assessed. A property is misplaced, if it is used as the object of a subject-predicate-object triple. There is an exception in which a property may be the object of defining triples whose property is used to define terms, such as ‘rdf:type’ and ‘rdfs:subPropertyOf’ (the ‘misplaced classes or properties’ metric).
9. The properties with a correct role in the triples are investigated whether they are ‘owl:dataTypeProperty’ or ‘owl:ObjectProperty’. The ‘owl:dataTypeProperty’ is misused if the related object is a URI; The ‘owl:ObjectProperty’ is misused if the related object is a literal (the ‘misuse of owl:dataTypeProperty or owl:ObjectProperty’ metric).
10. An assessment report is generated, which contains all the assessment results: a list of all errors (e.g., non-resolvable, non-parsable URIs) and their proportions to all unique URIs. The number of triples affected and their percentage is calculated for quantitative analysis.

**Table 2:** Mappings between RDF serialization formats and Common Media types. Adapted from [8]

RDF serialization format	RDF content-type
Turtle	text/turtle, application/x-turtle
N-Triples	text/plain
JSON-LD	application/ld+json
Notation 3	text/n3
RDF/XML	application/rdf+xml
RDF/JSON	application/ld+json

The quality assessment of RDF resources following the workflow on the aforementioned metrics (see Table 1) was implemented on December 30th 2022 in an open-source tool available on GitHub [14], which was written in Python using the rdflib package [15].

### Semantic representation of assessment results

**Fig. 3:**
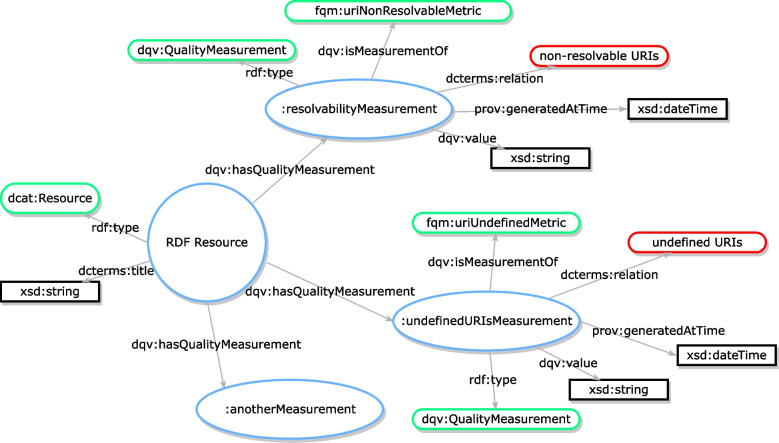
The structure of an assessment report. In this model, the RDF resource is of type ‘dcat:Resource’ and connects all quality measures. Each quality measure is a node (i.e., URI) that connects its metadata, such as the metric it is measured against using ‘dqv:isMeasurementOf’, the date and time it is generated using ‘prov:generatedAtTime’, and the erroneous URIs identified using ‘dcterms:relation’. All of the node names in this diagram are examples for illustrative purposes

The quality of a resource is a valuable piece of metadata that reflects that resource’s trustworthiness and enables the efficient filtering of high-quality resources. To facilitate the sharing of quality information, we represented assessment results of RDF resources using the quality model of the Data Quality Vocabulary (DQV) [16]. Figure 3 depicts the whole adapted version of the quality model to our situation.

To demonstrate the benefits of utilizing this semantic representation for assessment reports, we formulated three questions to be answered through SPARQL querying [17]:

1. Which rare-disease resources have more than 10% non-resolvable URIs?
2. Which rare-disease resources have undefined URIs? Which are these undefined URIs?
3. What quality issues are identified in the WikiPathways WP Ontology? Which metrics are they referring to? What is the definition of these metrics?

This demonstration process was implemented in Ontotext GraphDB [18], an RDF triplestore that provides a SPARQL endpoint.

## Results

### Assessment results

Sixteen rare-disease resources were selected, as shown in Table 3, including 2 schemas, 3 metadatasets, and 11 ontologies. Their basic characteristics are shown in Table 4, including their number of URIs, literals, blank nodes, and RDF triples in them. Additionally, the running time to perform the quality assessment is shown.

**Table 3:** Overview of selected RDF resources in the domain of rare diseases

Resource	Description	Version	Link
rare-disease biobanks and registries	Metadataset providing a list of biobanks and patient registries in the EJP RD.	2021/9/30	https://doi.org/10.5281/zenodo.8430384
Head and neck tumor registry Austria	Metadata of a single patient registry.	2021/9/30	https://doi.org/10.5281/zenodo.8413872
A biobank of patients with Primary Immune Deficiencies	Metadata of a single biobank.	2021/9/30	https://doi.org/10.5281/zenodo.8430367
NeXtProt schema	A schema describing the structures of NeXtProt data.	2022/6/28	https://download.nextprot.org/pub/current_release/rdf/ttl/schema.ttl.gz
Orphanet catalog schema	A schema describing the ontological structure of proof-of-concept FAIR Data Point for biobank and patient registry in the EJP RD.	2022/6/14	https://doi.org/10.5281/zenodo.8430340
hPSCreg vocabulary	A collection of terms used in the registry for human pluripotent stem cell lines ( hPSC lines).	2022/7/26	https://hpscreg.eu/ontologies/
Resource Metadata Ontology	A collection of terms used in the EJP RD metadatasets.	v 1.0	https://raw.githubusercontent.com/ejp-rd-vp/resource-metadata-schema-ontology/main/ejprd_resource_metadata_ontology.owl
The WikiPathways Ontology	Ontology defining the classes and properties used in the WikiPathways - a collaborative platform for the creation and maintenance of content related to biological pathways.	2022/7/11	https://vocabularies.wikipathways.org/wp
NeXtProt vocabulary	Ontology defining all the classes and properties used in the NeXProt - an online knowledge platform on human proteins.	2022/6/28	https://download.nextprot.org/pub/current_release/rdf/ttl/terminology.ttl.gz
UniProt ontology	A collection of terms used to describe the entries and associated data in UniProt, which is the database of protein sequence and functional information.	2022/3/1	http://purl.uniprot.org/core/
Orphanet Rare Disease Ontology (ORDO)	‘A structured vocabulary for rare diseases derived from the Orphanet database, capturing relationships between diseases, genes and other relevant features.’	2022/12/6	https://www.orphadata.com/ordo/
Anatomical Therapeutic Chemical Classification (ATC)	‘a classification of active ingredients of drugs according to the organ or system on which they act and their therapeutic, pharmacological and chemical properties.’	2022/5/2	https://bioportal.bioontology.org/ontologies/ATC
Human Phenotype Ontology (HPO)	‘a standardized vocabulary of phenotypic abnormalities encountered in human disease.’	2022/10/5	http://purl.obolibrary.org/obo/hp/releases/2022-10-05/hp.owl
Gene Ontology (GO)	A knowledge base providing the information on the functions of genes.	2022/8/10	https://bioportal.bioontology.org/ontologies/GENO
SNOMED CT	‘It is the most comprehensive, multilingual, clinical healthcare terminology in the world and is a resource with scientifically validated clinical content that is released monthly.’	2020/3/11	Not available. License is needed.
National Cancer Institute Thesaurus (NCIT)	‘It is a widely recognized standard for biomedical coding and reference, used by a broad variety of public and private partners both nationally and internationally’	01/05/2023	https://data.bioontology.org/ontologies/NCIT/download?apikey=8b5b7825-538d-40e0-9e9e-5ab9274a9aeb &download_format=rdf

**Table 4:** Basic characteristics of the sixteen RDF resources in the domain of rare diseases, including count of URIs, literals, and triples. Also the time to perform quality assessment on the resource is included (hour:minute:second)

Resource	# URI	# Literal	# Triple	Time cost
Orphanet catalog schema	46	11	39	00:01:25
rare-disease biobanks and registries	1,068	10	2,085	00:27:23
Head and neck tumor registry Austria	54	10	47	00:02:04
A biobank of patients with Primary Immune Deficiencies	46	10	38	00:02:01
Resource Metadata Ontology	257	721	1627	00:10:09
The WikiPathways Ontology	124	17	149	00:10:32
hPSCreg vocabulary	943	0	1,000	00:25:02
NeXtProt schema	895	1,410	3,291	00:04:07
NeXtProt vocabulary	269,987	264,549	1,188,696	14:50:25
UniProt ontology	396	4	391	00:07:35
ORDO	15,070	1043,03	1,142,401	02:42:05
ATC	6,712	18,993	66,682	02:13:22
GO	722	1,901	4,883	00:31:10
HPO	39,161	230,778	1,084,804	15:46:23
SNOMED CT	356,548	944,485	6,541,868	277:08:30
NCIT	174,590	1,224,526	8,775,164	98:23:20

After assessing these resources, the test revealed quality issues on three metrics: ‘non-resolvable URIs’, ‘undefined URIs’, and ‘misused owl:ObjectProperty or owl:dataTypeProperty’, as shown in Table 5.

**Table 5:** The count (#) and percentage (%) of errors identified and affected triples. Assessed on December 30th 2022. The remaining three metrics are not included as no quality issues are identified in these metrics. ^a^ This resource is a special case and is described in the Discussion

Resource	Non-resolvable URIs		Undefined URIs		Misused owl:ObjectProperty	
	URIs (#/%)	affected triples (#/%)	URIs (#/%)	affected triples (#/%)	URIs (#/%)	affected triples(#/%)
rare-disease biobanks and registries	5/1,068 (0.5%)	1,039/2,085 (49.8%)	0	0	0	0
Head and neck tumor registry Austria	6/54 (11.1%)	10/47 (21.3%)	0	0	0	0
A biobank of patients with Primary Immune Deficiencies	5/46 (10.9%)	9/38 (23.7%)	0	0	0	0
NeXtProt schema	0	0	1/895 (0.1%)	1/3,291 (0.0%)	0	0
Orphanet catalog schema	4/46 (8.7%)	13/39 (33.3%)	0	0	0	0
hPSCreg vocabulary	95/943 (10.1%)	105/1,000 (10.5%)	1/943 (0.1%)	1/1,000 (0.1%)	0	0
Resource Metadata Ontology	26/257 (10.1%)	71/1,627 (4.4%)	2/257 (0.7%)	4/1,627 (0.2%)	0	0
The WikiPathways Ontology	17/124 (13.7%)	87/149 (58.4%)	1/19 (0.8%)	1/149 (0.7%)	1/19 (5.3%)	2/149 (1.3%)
NeXtProt vocabulary	0	0	0	0	0	0
The UniProt ontology	0	0	2/396 (0.5%)	2/391 (0.5%)	0	0
ORDO	53/15,070 (0.3%)	162,684/1,142,401 (14%)	0	0	0	0
ATC	6/6,712 (0.1%)	14,446/66,682 (21.7%)	0	0	0	0
HPO	300/39,161 (0.8%)	17,870/1,084,804 (1.6%)	60/39,161 (0.2%)	1,855/1,084,804 (0.2%)	1/88 (1.1%)	2/1,084,804 (0.0%)
GO	7/722 (0.9%)	23/4,883 (0.5%)	2/722 (0.2%)	7/4,883 (0.1%)	0	0
SNOMED CT ^a^	356,523/356,548 (99.9%)	6,541,865/6,541,868 (99.9%)	0	0	0	0
NCIT	0	0	0	0	0	0

Except for four resources that have no non-resolvable URIs (i.e., NeXtProt schema, NeXtProt vocabulary, UniProt ontology, and NCIT) and SNOMED CT, which is a special case, as is addressed in the Discussion, the proportion of non-resolvable URIs in the remaining eleven resources ranges from 0.1% (6/6,712) in ATC to 13.7% (17/124) in the WikiPathways Ontology. These non-resolvable URIs have affected multiple triples within the resources, ranging from 0.5% (23/4,883) in GO to 58.4% (87/149) in the WikiPathways Ontology. Ninety-nine percent of URIs from SNOMED CT are non-resolvable and they all are the terms defined by SNOMED CT. The remaining 1% resolvable URIs are the terms from SKOS [19](e.g., <http://www.w3.org/2004/02/skos/core#definition>), OWL [20] (e.g., <http://www.w3.org/2002/07/owl#versionIRI>), and RDFS [21] (e.g., <http://www.w3.org/2000/01/rdf-schema#label>).

Seven out of the sixteen resources use URIs that are undefined: NeXtProt schema (1), hPSCreg vocabulary (1), Resource Metadata Ontology (2), the WikiPathways Ontology (1), the UniProt ontology (2), HPO (60), and GO (2), see Table 6 for examples of such undefined URIs.

**Table 6:** Examples of undefined URIs

Resource	Undefined URI	Comment
NeXtProt schema	http://www.w3.org/2002/07/owl#	http://www.w3.org/2002/07/owl (without hashtag) is defined.
hPSCreg vocabulary	http://www.w3.org/2000/01/rdf-schema#source	‘rdfs:source’ does not exist but ‘rdfs:Resource’ exists.
Resource Metadata Ontology	http://www.w3.org/ns/prov-o	It points to the RDF representation of PROV ontology, while the prefix of this ontology is http://www.w3.org/ns/prov#
	http://www.w3.org/ns/prov-o-20130312	Version URI, resolving to the same content as above.
The WikiPathways Ontology	http://purl.org/dc/terms/accuralPeriodicity	Typo. It should be ’accrualPeriodicityMore’.
The UniProt ontology	http://www.w3.org/1999/02/22-rdf-syntax-ns	http://www.w3.org/1999/02/22-rdf-syntax-ns# (with hashtag) is defined.
Human Phenotype Ontology (HPO)	http://purl.org/dc/elements/1.1/license	Mix of DCMI namespaces. It should be ‘http://purl.org/dc/terms/license’.
Gene Ontology (GO)	https://doi.org/10.1186/s13326-017-0126-0	Every Digital Object Identifier (DOI) is not ’defined’ in a machine-readable way.

Only WikiPathways Ontology and HPO have shown inconsistency between the owl:ObjectProperty property and the linking object that should be a URI (i.e., other resources) rather than a literal (e.g., string, integer). The triples including these properties are shown in Table 7. Although the metric ‘misuse of owl:dataTypeProperty or owl:ObjectProperty’ literally focuses on properties, it can also indicate that the object of the property causes the inconsistency. For example, the object ‘http://purl.obolibrary.org/obo/MI_0915’ (with quotes) is recognized as ‘string’ by computers and therefore of incorrect type, whereas <http://purl.obolibrary.org/obo/MI_0915> is recognized as a URI. In this instance, the object, not the property, causes the inconsistency.

**Table 7:** Identified properties of owl:ObjectProperty type with triples

Resource	Subject	Misused owl:ObjectProperty	Object
The WikiPathways Ontology	http://data.wikipathways.org/20220410/rdf/wp	http://www.w3.org/ns/dcat#mediaType	‘application/zip’
Human Phenotype Ontology	http://purl.obolibrary.org/obo/RO_0002436	http://www.w3.org/2004/02/skos/core#closeMatch	‘http://purl.obolibrary.org/obo/MI_0915’

### Assessment reports

**Fig. 4:**
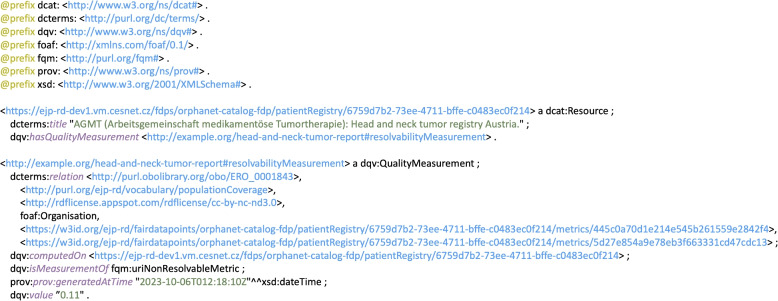
An example assessment report for AGMT head and neck tumor registry in Austria

Conforming to the DQV quality model, sixteen assessment reports in Turtle serialization format were generated upon the completion of the rare-disease resources assessment procedure. Figure 4 depicts the report for the resource describing the metadata of the AGMT (Austrian Group Medical Tumor Therapy) head and neck tumor registry in Austria. It indicates that this RDF resource failed only one metric, namely the ‘non-resolvable URIs’ metric. This quality measure is related to six URIs (see the ‘dcterms:relation’ triples), meaning that these are the six URIs classified by the tool as non-resolvable. As indicated by the ‘dqv:value’ attribute, their proportion is ‘6/54’.

**Fig. 5:**
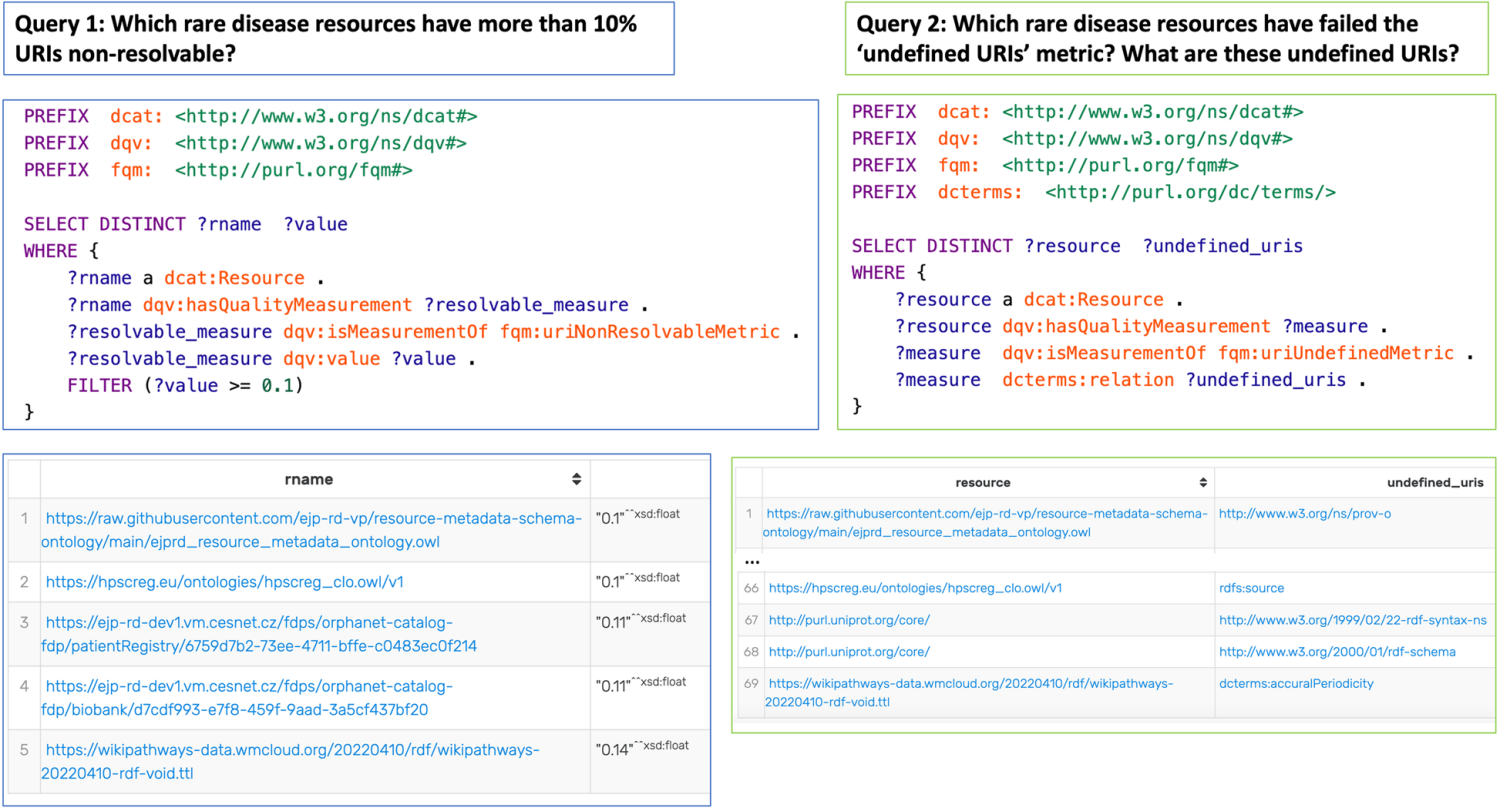
Two SPARQL queries with their results to answer the first two questions. The query to answer the third question is available in Additional file 1

As depicted in Fig. 5, three SPARQL queries were generated to answer the proposed questions to demonstrate the added value of semantic representation of assessment reports.

Which rare-disease resources have more than 10% non-resolvable URIs? Five resources contain over 10% non-resolvable URIs. This is answered by first querying the quality measurements that are based on the ‘fqm:uriNonResolvableMetric’, and then filtering the recorded value according to the ‘dqv:value’ property.

Which rare-disease resources have undefined URIs? Which are these undefined URIs? There are 69 undefined URIs with corresponding resources listed in the query results. This is answered by first querying the quality measurements based on the ‘fqm:uriUndefinedMetric’, and then retrieving all the URIs following the ‘dcterms:relation’ property.

What quality issues are identified in the WikiPathways WP Ontology? Which metrics are they referring to? What is the definition of these metrics? There are three types of quality issues identified. In the query results, their corresponding metrics with definitions are displayed. This is answered by querying for existing quality measurements with their metrics, and then retrieving the definitions following the ‘skos:definition’ property which is used in the FQM ontology.

## Discussion

In this paper, we applied six metrics to assess the quality characteristics of RDF resources in the rare-disease domain. We found a few issues when assessing the quality of these resources: eleven out of sixteen resources have non-resolvable URIs; seven resources have undefined URIs; two resources have inconsistency related to the ‘owl:ObjectProperty’ properties. Individual findings will be discussed in more depth in the sections that follow.

### Insights into errors

Numerous resources such as the ORDO used the property <https://creativecommons.org/licenses/permits> and the class <http://web.resource.org/cc/Attribution> to describe the Creative Commons licenses. However, both of these are non-resolvable. The correct ones are <https://creativecommons.org/ns#permits> and <https://creativecommons.org/ns#Attribution> [22]. This implies that there is a lack of up-to-date communication between ontology creators and the Creative Commons organization.

There are some URIs that are classified by the algorithm as ‘undefined’ that are actually ‘defined’, according to the definition of the ‘undefined URIs’ metric. For example, the URI <http://www.w3.org/ns/prov-o> in the EJP RD Resource Metadata Ontology (see Table 6) is described in the triple: <http://www.w3.org/ns/prov-o#> rdf:type owl:Ontology.

Both URIs point to the same resource but are syntactically different (i.e., a URI with a hashtag compared to one without a hashtag). These examples show that any approach or technique based on pattern matching is heavily reliant on the accuracy of URIs. Also classified as ‘undefined’ are the other two ontology URIs <http://www.w3.org/1999/02/22-rdf-syntax-ns> and <http://www.w3.org/2000/01/rdf-schema> in the UniProt Ontology without hashtags. Another example is the URI <https://doi.org/10.1186/s13326-017-0126-0>. It is classified as ‘undefined’ in GO, because it does not exist in its triples that were parsed. The URI <http://dx.doi.org/10.1186/s13326-017-0126-0> is however defined. One should not use one URI for definition whilst using another URI for referencing it.

Besides, URIs whose ‘path’ part contains letters are more susceptible to any operation that is affected by case sensitivity. For example, ‘dcat:catalog’ (<http://www.w3.org/ns/dcat#catalog>) is a property while ‘dcat:Catalog” (<http://www.w3.org/ns/dcat#Catalog>) is a class. Their ‘path’ parts, ‘#catalog’ (lowercase) versus ‘#Catalog’ (upper case), are different. Such a small distinction makes it easy to confuse them. However, this issue can be alleviated by incorporating codes into the naming, for example, the ‘is located in’ property <http://semanticscience.org/resource/SIO_000061> and the class ‘female’ <http://purl.bioontology.org/ontology/SNOMEDCT/248152002> using only numbers.

Mismatched prefixes or terms are a common cause of undefined URIs. One example is <http://purl.org/dc/elements/1.1/license>, which is used in the HPO. It does not exist, whereas <http://purl.org/dc/terms/license> does exist, though both are resolvable. This is because two Dublin Core$^{\textrm{TM}}$ Metadata Initiative (DCMI) namespaces [23] were mixed up: ‘http://purl.org/dc/elements/1.1/’ and ‘http://purl.org/dc/terms/’. Another example is ‘rdfs:source’ (<https://www.w3.org/2000/01/rdf-schema#source>) used in the hPSCreg vocabulary. This term does not exist; however, ‘rdfs:Resource’ does. This is probably due to the misinterpretation of existing terms. Both examples demonstrate the need for automated quality assessment by machines to detect errors that are often hard to detect by humans.

Importantly, we do not regard a URI that does not have content-type RDF to be an error because such a URI already indicates that it does not provide an RDF representation. For instance, the URI <https://www.ietf.org/rfc/rfc3986.txt> with the ‘text/plain’ content-type in the ‘rare-disease biobank and registries’ resource and the URI <https://github.com/geneontology/go-ontology/issues/7549> with the ‘text/html’ content-type in HPO properly use non-RDF content. It is also essential to emphasize that the purpose of identifying errors in these resources is not to dissuade people from using them, but rather to suggest areas for improvement so that the rare-disease community can benefit from ‘linked data’ and RDF.

### Strengths and limitations

Our effort to assess the quality of RDF resources in the domain of rare diseases has several strengths. First of all, a significant strength is that the metrics applied are objective and automatable, allowing the quality assessment to be easily scalable when applied to other RDF resources while yielding reliable results. Secondly, the assessment report is generated in the form of RDF, allowing the quality information to be shared and reused in the future to accommodate the dynamic nature of resources in the world of Linked Data.

There are limitations in the implementation of the assessment of the metrics. First, the current evaluation tool relies on pattern matching and is limited to the syntactical level, therefore does not deem two URIs with and without hashtags as identical. Second, the current version of the tool does not adequately handle instances. One example is the URI <http://purl.obolibrary.org/obo/IAO_0000120> which stands for ‘metadata complete’ and is an instance of ‘curation status specification’ (i.e., <http://purl.obolibrary.org/obo/IAO_0000078>), i.e., defined as ‘owl:NamedIndividual’ rather than ‘owl:Class’ or ‘rdfs:Class’. Only the metrics regarding resolvability and parsability are applicable, so the tool only tested instances based on these two metrics. Nevertheless, it is necessary to include additional metrics that measure different aspects of instances, which should be the subject of future work. One example of a metric may be detecting an instance as a type of two disjoint classes, which can lead to inconsistency.

### Lessons learned for quality assessment

Given the size of biomedical ontologies, it is necessary to design the most computationally efficient methods prior to metric implementation in terms of memory consumption and time cost, especially for a large-sized ontology (e.g., NCIT with over 170,000 terms) or when an ontology server has a blocking mechanism to prevent repeated external requests. For example, the assessment of SNOMED CT revealed that all the URIs stemming from SNOMED CT (i.e., those starting with ‘http://snomed.info/’) return the status code 423 Locked. This is not a quality issue of these URIs but is attributed to a blocking mechanism, despite retry and sleep functions being applied in the software. Both functions again increase the total running time of implementation. To enable consistency assessment in this type of cases, one potential approach is to retrieve a complete RDF representation of the resource, such as an ontology, a schema, or a (meta)dataset, and make it available in a triplestore as a temporary RDF graph to be referred to by assessed URIs.

Even though the current quality model is adequate for representing the quality metadata in RDF, the more resources are investigated, the more amendments or extensions may be required. DCAT <https://www.w3.org/ns/dcat#>, for instance, supports multiple RDF serialization formats, such as JSON-LD and Turtle. DCAT in JSON-LD <https://www.w3.org/ns/dcat2.jsonld> and Turtle <https://www.w3.org/ns/dcat2.ttl> are likely to produce different data quality measures, due to the fact that the graphs parsed from both URIs are not identical. A potential solution to address it is to treat (resources in) each serialization format as an individual resource and link the quality measures to the particular format assessed. Through the property ‘dcat:distribution’, each (resource in) serialization format can be linked to the original resource URI, such as DCAT <https://www.w3.org/ns/dcat#>.

### Recommendation for creation of high-quality rare-disease resources

In this paper, we consider a resource to be of high quality if it does not have any foundational quality issues. Although some [16, 24] argue that resource quality is subjective and in the eye of the beholder, the foundational quality aspects emphasized in this work remain objective and fundamental for all resources. Here are some recommendations learned from this study for the creation of high-quality RDF resources in the domain of rare diseases:

- **Non-resolvable URIs:** (1) If one creates URIs, ensure that they are resolvable. Non-resolvable URIs need to be corrected and all URIs need to be tested periodically. (2) Avoid using URIs from external resources that are non-resolvable. Even if within a commonly-used ontology such as the ORDO, there are 42 non-resolvable URIs, which are used to describe rare-disease conditions.
- **Undefined URIs:** (1) If one creates URIs, it is recommended to only include digits in their naming so that they are case insensitive [25]. (2) If one reuses URIs from external resources, make sure to comprehend their namespaces and apply them correctly. Keep in mind that URIs of terminology may be case-sensitive, which can result in different resources being referenced when the capitalization of URIs is altered.
- **Inconsistent URIs:** (1) If one creates classes or properties, ensure that they adhere to intrinsic characteristics as ‘owl:Class’ or ‘rdfs:Property’, ‘owl:ObjectProperty’ or ‘owl:DataTypeProperty’; (2) If one reuses existing classes or properties, ensure that they adhere to the same intrinsic characteristics and that they are not deprecated.

### Related work and future work

There are some studies that investigated the quality issues related to foundational quality. Johannes et al. [26, 27] highlighted that the availability of (terms of) ontologies could significantly influence the reusability of resources that reference these ontologies. They conducted the ontology accessibility study on 1,439 ontologies on the DBpedia Archivo [28] platform, and found that 709 (46%) of these ontologies were not accessible at least once. Being inaccessible means that the ontology URI and all URIs defined in ontologies were non-resolvable, and they found that these non-resolvable ontologies have impacted 32% of linked data on the same platform. This finding based on ontologies on the Archivo platform is in line with our findings based on the rare-disease resources (including ontologies), indicating that non-resolvable URIs continue to be a problem in the Semantic Web community. Such a problem should be ‘resolved’, given the important role of identifiers in making data Findable, Accessible, Interoperable, and Reusable [29–32]. Identifiers (e.g., URIs) can make it easier to find resources in an unambiguous manner (F), ensure reliable access if resolvable and authorized (A), enable databases and repositories to recognize and computers to interpret the referred resources (I), altogether contributing to the reuse of resources (R).

Given the objective and automatable nature of the foundational quality metrics, it will be necessary in the future to assess resources in other domains to identify more quality issues in the real world, and accordingly to develop domain-specific guidelines.

## Conclusion

We assess the resolvability, parsability, and consistency of RDF resources in the rare-disease domain, and identify various types of errors. Using non-resolvable URIs is the primary quality issue, and there are numerous causes for undefined URIs. Based on the findings, recommendations regarding URIs have been provided. In the future, it will be necessary to incorporate more real-world scenarios to enable the assessment of resources from more diverse sources. Potentially, the applied methods for quality assessment can be integrated into the process of generating RDF resources, thereby enabling real-time quality assurance as opposed to post-hoc assessment and curation.

### Supplementary Information

**Additional file 1.** The SPARQL query with the result to answer the third proposed question.

## Data Availability

All the data assessed are open resources available on the Web, see their links in Table 3. The SPARQL queries are available at https://github.com/sxzhang1201/assess-rdf-resource/tree/master/query. All the assessment reports are compiled together and available at https://w3id.org/rdfqar.

## Abbreviations

URI(s): Unique Resource Identifier(s)
RDF: Resource Description Framework
OWL: Web Ontology Language
RDFS: RDF Schema
DQV: Data Quality Vocabulary
FQM: Foundational Quality Metrics
HPO: Human Phenotype Ontology
GO: Gene Ontology
ATC: Anatomical Therapeutic Chemical Classification
NCIT: National Cancer Institute Thesaurus
SPARQL: SPARQL Protocol and RDF Query Language
