# Supplementary figures and images for: Assessing resolvability, parsability, and consistency of RDF resources: a use case in rare diseases
Source: J Biomed Semantics. 2023 Dec 5;14:19. doi: 10.1186/s13326-023-00299-3 (PMC10696869; doi:10.1186/s13326-023-00299-3)

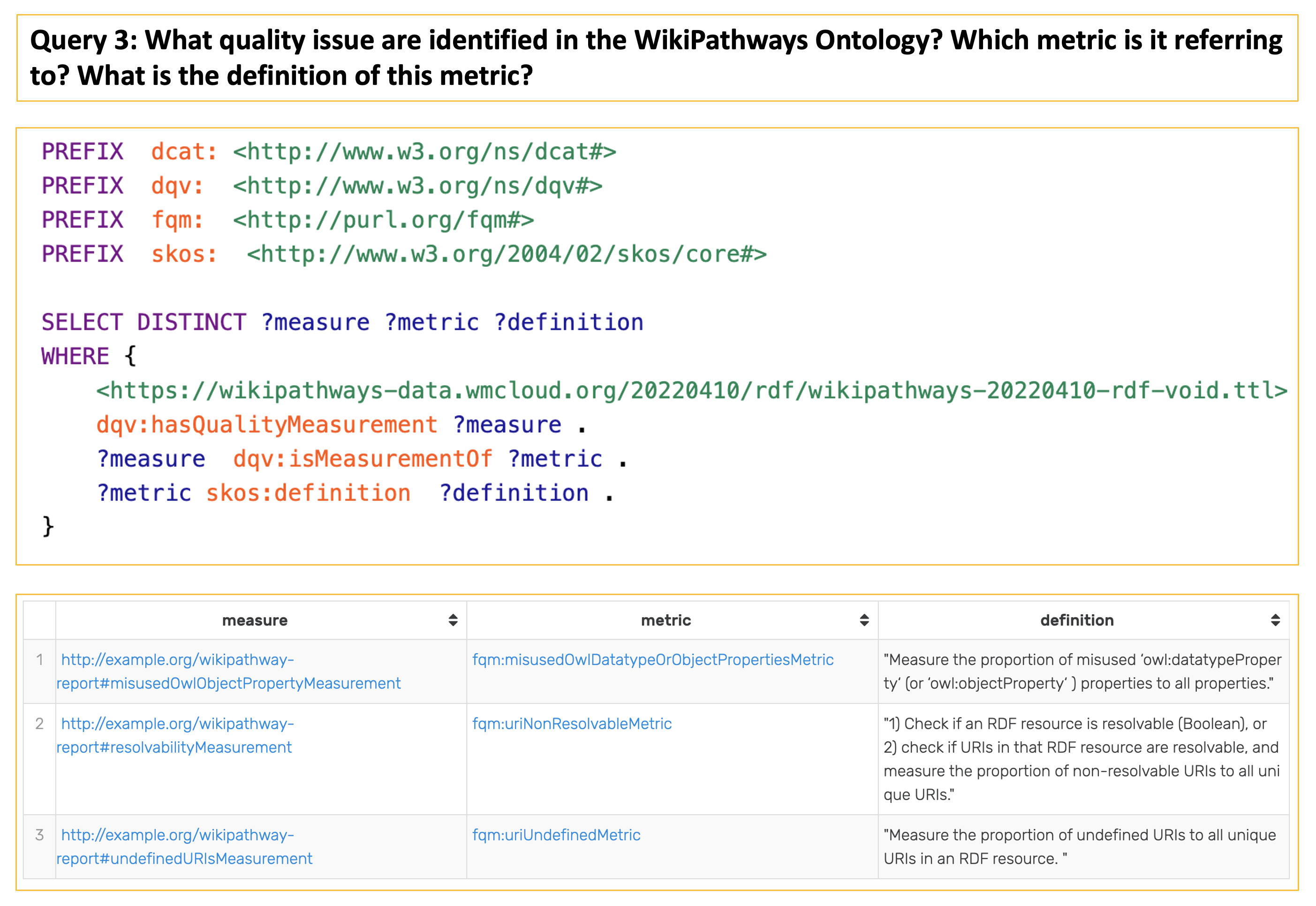

Supplement: Supplementary file 1 — Additional file 1. The SPARQL query with the result to answer the third proposed question. [file 13326_2023_299_MOESM1_ESM.jpg]
